# Intraosseous Lipoma of the Sphenoid: A Case Study

**DOI:** 10.1155/2013/519341

**Published:** 2013-05-28

**Authors:** Zygmunt Jamrozik, Grzegorz Rosiak, Biruta Kierdaszuk, Krzysztof Milczarek, Anna Kamińska, Dorota Dziewulska, Antoni Krzeski

**Affiliations:** ^1^Department of Neurology, Medical University of Warsaw, Banacha 1A Street, 02-097 Warsaw, Poland; ^2^Department of Radiology, Medical University of Warsaw, Poland; ^3^Department of Otolaryngology, Czerniakowski Hospital, Warsaw, Poland

## Abstract

Intraosseous lipoma is very rare, usually benign tumor of flat bones. However, the localization in skull bones is described in sporadic cases. The differential diagnosis includes end stage of infection, infarct lesions, intraosseous meningioma, angiolipoma, or myxofibrous tumors. We report a patient with intraosseous lipoma located in the sphenoid bone. The diagnosis was established due to the characteristic radiological features. According to the history of seizures, the lesion was removed via endoscopic endonasal approach. Histopathological examination showed adipocytes. The patient underwent control neuroimaging studies.

## 1. Introduction

 Intraosseous lipoma (IL) is a rarely diagnosed, usually benign tumor placed in the flat bones, hardly ever in the long bones. Most often reported localizations include calcaneus, rib, and frontal and basal skull [1–3]. Lipoma originates from mesenchymal tissue and might resemble other benign tumors. Incidence of sphenoid bone and sphenoid sinus lipoma seems to be very rare although precise epidemiological data are not known [4–6]. It has been postulated that intraosseous lipomas grow as a result of infarct, end stage of infection, fibrous dysplasia, fibro- and angiolipoma, or liposclerosing myxofibrous tumors (LSMFT). All those should be included in the differential diagnosis of the intraosseous lipoma.

## 2. Case Report

**Figure 1:**
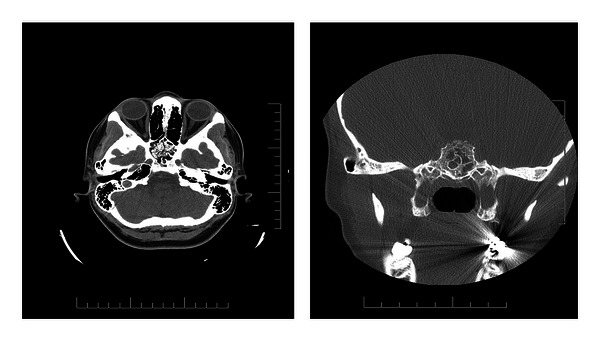
Routine CT scan revealed intraosseous lipoma within the sphenoid bone and sinus with small intratumor calcifications.

**Figure 2:**
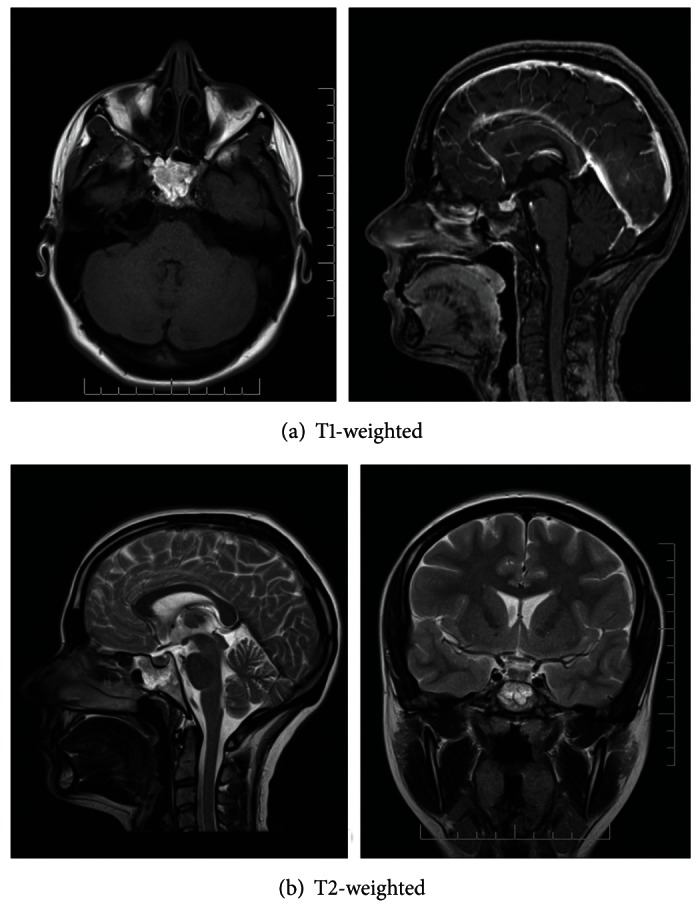
MR scans showed a high signal intensity in sphenoclival mass in both T1WI (SE) and T2WI (TSE) with sharp and thin margin of low signal intensity.

A patient, 23-year-old woman, has been referred to neurological department by an ophthalmologist with suspected papilledema. Two weeks earlier, she suffered from the first generalized tonic-clonic seizure after unexpected sudden pain. Medical history includes 2 attacks of unconsciousness after acute pain. A CT scan revealed a hypodense mass with central calcifications within sphenoid sinus and clivus. Density of the hypodense portion of mass ranged from −30 to −90 Hounsfield units suggesting presence of fat which surrounded central, scattered calcifications. Multilobulated margins of the lesion were sharp and sclerotic suggesting nonaggressive growth (Figure 1). MR scans showed a sphenoclival mass hyperintense in both T1WI (SE) and T2WI (TSE) with sharp and thin margin of low signal intensity corresponding with sclerotic rim. Cortex of sella turcica and clivus were intact. Fat suppressed sequence confirmed fat containing lesion; incomplete signal suppression was due to central calcifications. The lesion showed no contrast enhancement (Figure 2). Upon findings in both imaging modalities, a diagnosis of intraosseous lipoma was made.

**Figure 3:**
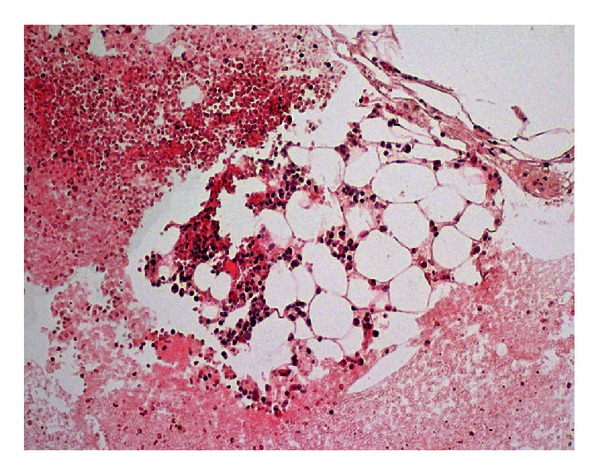
Fragment of the surgically removed tissues-visible conglomerates of adipose cells; hematoxylin and eosin staining.

Clinical neurological examination was normal except for a bilateral Babinski sign. All biochemical tests were normal, as well as EEG pattern. Patient has not been treated with antiepileptic drugs due to the first seizure and finally unrecognized cause of the epileptic event; besides that, reflex epilepsy was taken into account. Cerebrospinal fluid (CSF) examination revealed increased level of protein (59 mg%). Other components of the CSF were normal. MRI of the brain and angio-MRI were normal. Cerebral intravenous thrombosis was excluded. A month later, she was admitted to the otolaryngology department and an intraosseous mass was removed via endoscopic endonasal approach. Histological examination revealed only adipocytes (Figure 3). Three months after the first hospitalization, the patient was reexamined neurologically. She had no complaints and no neurological abnormalities, and no seizure was reported. Control MRI revealed a similar pattern but restricted to the sphenoid bone. 

## 3. Discussion

The presented case with radiological and clinical picture resembles cases described by MacFarlane et al. and Lanišnik et al. [7, 8]. According to Milgram classification, our patient belongs to stage III with focal calcification and cystic degeneration but without bone expansion and bone destruction [9, 10]. Recent reports revealed that sphenoid localisation is rare but was not so, as it was previously suggested [1, 4, 11, 12]. Intraosseous lipoma represents 0,1% of the primary bone tumours. Skull location of lipoma is estimated as about 4% of all of them but the incidence of the sphenoid localisation has not been established [12].

Recently, noninvasive followup has been proposed in case of the intraosseous lipoma due to low possibility of transforming into malignant forms, a relatively precise diagnosis based on neuroimaging tests and sometimes spontaneous involution [11, 13–15]. 

Our patient underwent invasive procedure because of the additional neurological symptoms as seizure and elevated CSF protein level. However, the relation of these symptoms to lipoma seems to be coincidental.
